# Isolation of Phytochemicals from *Bauhinia variegata* L. Bark and Their In Vitro Antioxidant and Cytotoxic Potential

**DOI:** 10.3390/antiox8100492

**Published:** 2019-10-17

**Authors:** Neha Sharma, Anket Sharma, Gaurav Bhatia, Marco Landi, Marian Brestic, Bikram Singh, Jatinder Singh, Satwinderjeet Kaur, Renu Bhardwaj

**Affiliations:** 1 Department of Botanical and Environmental Sciences, Guru Nanak Dev University, Amritsar 143005, India; nehasharma.env@gmail.com (N.S.); anketsharma@gmail.com (A.S.); sjkaur2001@yahoo.co.in (S.K.); 2 State Key Laboratory of Subtropical Silviculture, Zhejiang A&F University, Hangzhou 311300, China; 3 Department of Biophysics, Panjab University, Chandigarh 160014, India; gauravbhatia22@gmail.com; 4 Department of Molecular Biology and Biochemistry, Guru Nanak Dev University, Amritsar 143005, India; jatinderarora2009@gmail.com; 5 Department of Agriculture, Food and Environment, University of Pisa, 56124 Pisa, Italy; 6 Department of Plant Physiology, Faculty of Agrobiology and Food Resources, Slovak University of Agriculture, Nitra 94976, Slovakia; marian.brestic@uniag.sk; 7 Department of Botany and Plant Physiology, Faculty of Agrobiology, Food and Natural Resources, Czech University of Life Sciences, 16500 Prague, Czech Republic; 8 Natural Product Chemistry and Process Development Division, CSIR-Institute of Himalayan Bioresource Technology, Palampur, Himachal Pradesh 176061, India; bikram_npp@rediffmail.com

**Keywords:** antioxidative system, anti-cancer compounds, oxidative stress, polyphenols, phytochemicals

## Abstract

Plants have been the basis of traditional medicine since the dawn of civilizations. Different plant parts possess various phytochemicals, playing important roles in preventing and curing diseases. Scientists, through extensive experimental studies, are playing an important part in establishing the use of phytochemicals in medicine. However, there are still a large number of medicinal plants which need to be studied for their phytochemical profile. In this study, the objective was to isolate phytochemicals from bark of *Bauhinia variegata* L. and to study them for their antioxidant and cytotoxic activities. The bark was extracted with methanol, followed by column chromatography and thus isolating kaempferol, stigmasterol, protocatechuic acid-methyl ester (PCA-ME) and protocatechuic acid (PCA). 2,2-azinobis-3-ethyl-benzothiazoline-6-sulfonic acid (ABTS) and 2, 2’-diphenyl-1-picrylhydrazyl radical (DPPH) radical scavenging assays were utilized for assessment of antioxidant activity, and 3-(4,5-dimethylthiazol-2-yl)-2,5-diphenyl tetrazolium bromide (MTT) dye reduction assay was used to determine cytotoxic activity against C-6 glioma rat brain, MCF-7 breast cancer, and HCT-15 colon cancer cell lines. The compounds were found to have significant antioxidant and cytotoxic activity. Since there is a considerable increase in characterizing novel chemical compounds from plant parts, the present study might be helpful for chemotaxonomic determinations, for understanding of medicinal properties as well as for the quality assessment of herbal supplements containing *B. variegata* bark, thus establishing its use in traditional medicine.

## 1. Introduction

Environmental factors like polluted water, air, soil, UV radiations, intake of pesticides by food consumption, all might result in production of reactive oxygen species (ROS) [1,2,3,4]. Imbalance between ROS production and their scavenging by antioxidant apparatus cause oxidative stress which is the base of damages to cell structure, proteins, lipids, and DNA [5,6,7]. A number of transcription factors are activated via ROS, which results in expressing proteins regulating inflammation, survival of tumor cells, cellular transformation, proliferation and invasion, metastasis and angiogenesis [8]. The disturbance of the homeostasis of cellular machinery caused by ROS leads to various pathological disorders, among which cancer is doubtless the most serious and worrying. Cancer has become a public health concern in developed as well as developing countries on a massive level. In India, it is a major cause of adult deaths, with more than 70% of lethal cancers occurring in people in the productive ages of 30–69 years [9]. The World Cancer Report 2014, launched by The International Agency for Research on Cancer (IARC), tells that the worldwide burden of cancer has reached to approximately 14 million new cases annually, and this will further rise to around 22 million per year in next two decades. Further, cancer deaths have been expected to rise from 8.2 million (approximately) to 13 million every year [10].

Among other classes of health promoting compounds, polyphenols from fruits, vegetables and medicinal plants represent a peerless class of antioxidants which can counteract ROS-triggered damages. The cancer preventive activities of various phytochemicals lie in their ability to affect cellular defenses like detoxification, scavenging ROS, as well as induction of antitumor and anti-inflammatory responses. Thus, antioxidants from medicinal plants present themselves as the safer option to chemically formulated anticancer drugs [11,12,13]. A huge diversity of polyphenol structures are produced by plants, and along with contributing to the color, flavor, odor, bitterness and astringency of food, these possess several biological activities like anti-allergic, anti-microbial, anti-viral, antioxidant, anti-inflammatory, anti-mutagenic, and anti-proliferative [14].

Prevention is better than cure, and cancer, though being a major cause of deaths worldwide, is a disease which can largely be prevented by modulating dietary supplements [15]. Among the reported cancer cases, 5–10% are linked to genetics and the other 90–95% cancers originate from various environmental and lifestyle reasons and are preventable [16]. The practice of using medicinal plant parts has been demonstrated since ancient times to treat a wide array of illnesses [17] and it is the need of the hour to return to plant based diet and to re-evaluate the effectiveness of herbal treatments against several dreaded diseases. Also, synthetic drugs and the pharmaceutical products, being used in human and veterinary medicine have certain side effects and are an emerging class of environmental contaminants [18]. Even though polyphenols are prevalent in the medicinal plant parts, not all people worldwide can ensure an adequate intake due to different reasons. The extraction, identification and separation of the antioxidant polyphenolic compounds from various plant parts is undertaken to help manufacturing dietary antioxidants with effective influence to be used as supplements to the normal diet [19].

The genus *Bauhinia* belongs to family Fabaceae and it includes an approximate of 350 shrubs, lianas and small tree species which are recognized easily with their bilobed leaves as well as palmate venation. Due to its bilobed leaves, the genus was named *Bauhinia* by Linnaeus to honor Swiss Botanists, Johann and Gaspard, who were identical twins [20]. The genus is broadly distributed in countries in Asia, South America, and Africa. Plant parts are often used in traditional medicine for infections, inflammations and diabetes. *Bauhinia* species like *B. rufescens*, *B. reticulata*, are used traditionally for treatment of ulcers, roundworm infections, conjunctivitis, dysentery, blood-poisoning, lung and skin diseases [21,22]. *Bauhinia variegata* Linn., a medium sized, deciduous tree, is also known as Kanchanara in Sanskrit; Kovidara in Hindi and Raktakanchan in Marathi. Almost all the parts find uses inside the traditional medicine against various ailments like leprosy, piles, asthma, ulcer, liver complaints and snake bite. It also finds its uses in the treatment of skin diseases, wound healing, obesity, stomatitis, dyspepsia, flatulence and is used as tonic, astringent and laxative as well [23]. An alcoholic extract of *B. variegata* stem bark has shown significant hepatoprotective activity against liver damage caused by carbon tetrachloride [24]. The ethanolic extract of *B. variegata* stem has shown antitumor activity against Dalton’s Ascytic lymphoma and Ehrlich ascites carcinoma in swiss albino mice [25,26]. Isolation of quercetin, rutin, apigenin and apigenin 7-O-glucoside has been reported from the different plant parts of *B. variegata* [27]. Literature survey suggests that although *B. variegata* plant parts have many medicinal uses, however, few studies have been undertaken to validate the therapeutic claim in a scientific manner. To introduce *B. variegata* to the list of potential sources of chemo-preventive phytochemicals, the present work was undertaken for the isolation and characterization of bioactive compounds from bark extracts and to estimate their antioxidant and cytotoxic potential.

## 2. Materials and Methods

### 2.1. Study Material

The study material for the present experimentation included bark of ethnobotanically important plant *B. variegata* Linn. To collect the plant material, a disease free tree was identified from the Guru Nanak Dev University, Mata Kaulan Botanical Garden, Amritsar, India, and taxonomical identification was done by the Division of Botany, Forest Research Institute, India. A specimen with voucher no. 0391/Herb has been deposited in the herbarium of GNDU, India.

### 2.2. Extraction of B. variegata bark

**Figure 1:**
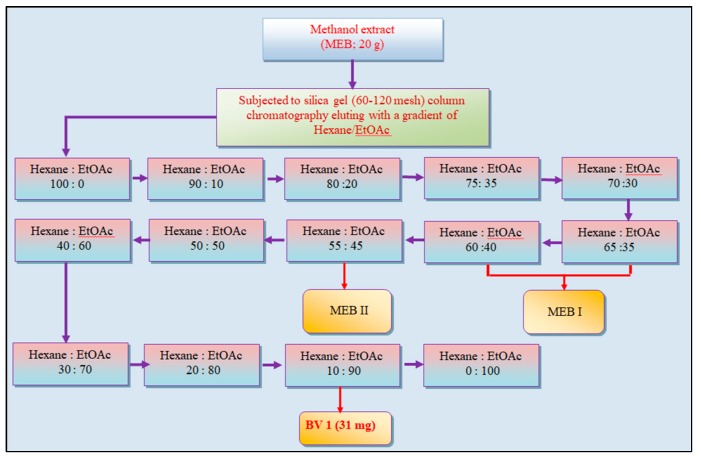
The schematic representation of column chromatography of methanol extract (MEB) of bark of *Bauhinia variegata* L. BV1 is the pure fraction obtained, subjected to further characterization. MEB I and MEB II are sub-fractions, subjected to further chromatography.

The stem bark of *B. variegata* L. (4.5 Kg) was washed, oven-dried at ±40 °C, and then powdered. It was percolated with 80% methanol and was dried under vacuum with rotary evaporator (Buchi R-210, Switzerland). The extract was further lypholized to get the methanol extract (MEB). The MEB extract of *B. variegata* bark was subjected to column chromatography. MEB extract (20 g) was then dissolved in 50 mL methanol and mixed with silica gel to make slurry. A column was packed (silica gel; 60–120 mesh) and slurry was loaded onto silica gel. Non-absorbant cotton was put on slurry to avoid any disturbance during column elution, which was done with a gradient of hexane/ethyl acetate (hex/EtOAc) (100:0), (90:10), (80:20), (75:35), (70:30), (65:35), (60:40), (55:45), (50:50), (40:60), (30:70), (20:80), (10:90), (0:100). 160 fractions (50 mL each) were collected and 0.2 mm thick Precoated Kieselgel F254-plates were used to carry out thin layer chromatography (TLC). The fractions eluted from column with hexane/ethyl acetate (10:90) yielded a yellow-colored compound, with a single spot in TLC, named BV1 fraction (31 mg) (Figure 1).

**Figure 2:**
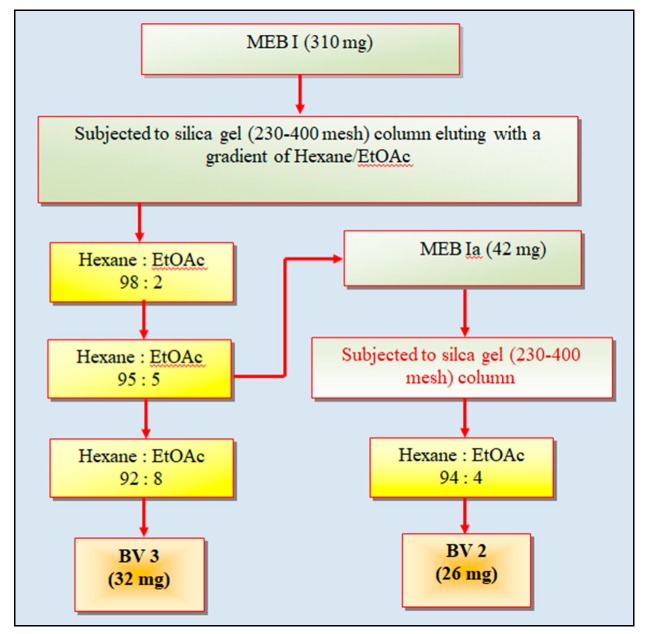
Column chromatography of fractions MEB1 and MEB1a obtained from column chromatography of methanol extract (MEB) of bark of *Bauhinia variegata* L. BV2 and BV3 are the pure fractions obtained, subjected to further characterization.

The fractions eluting in hexane/ethyl acetate (65:35) to hexane/ethyl acetate (60:40) (Figure 1) were pooled, based on identical spots on TLC. The pooled fractions were dried and the solid fraction MEBI (310 mg) was dissolved in ethanol (5 mL), mixed and dried with silica gel to make slurry and loaded on column packed with silica gel (230–400 mesh). The column was eluted with a gradient of hexane/ethyl acetate (98:2), (95:5), (92:8) and 15 fractions (15 mL each) were collected and the fractions from hexane/ethyl acetate gradient (95:5) were pooled based on TLC and precipitates were collected after leaving for overnight. These precipitates, named as MEBIa fraction (42 mg) were washed and eluted in a column with hexane/ethyl acetate (94:4), thus yielding white-colored compound named as BV2 fraction (26 mg). The fractions collected from hexane/ethyl acetate gradient (92:8) from column chromatography of MEBI were pooled and dried to yield a dark yellow-colored compound which was named as BV3 fraction (32 mg) (Figure 2).

**Figure 3:**
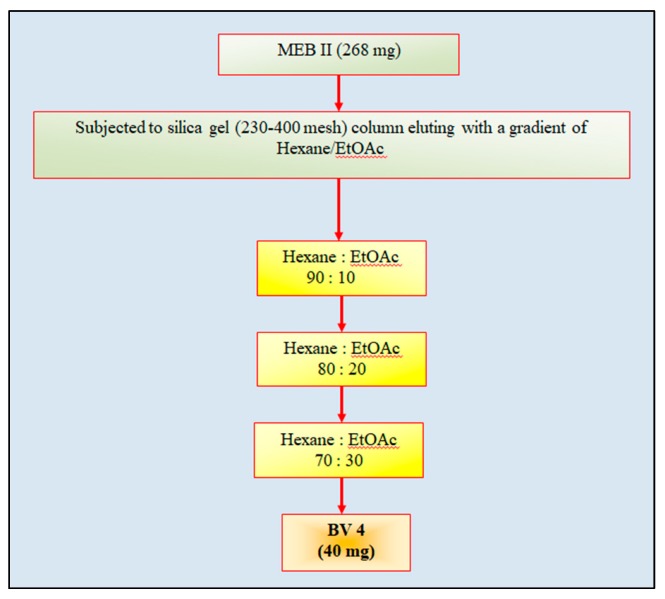
Column chromatography of fraction MEB II obtained from column chromatography of methanol extract (MEB) of bark of *Bauhinia variegata* L. BV4 is the pure fraction obtained, subjected to further characterization.

Five fractions eluting from column of MEB in hexane/ethyl acetate (55:45) (Figure 1)were pooled on basis of similar Rf on TLC and were dried. The dried fraction (MEBII; 268 mg), dissolved in ethyl acetate (5 mL), mixed and dried with silica gel to prepare slurry, was eluted in a column packed with silica gel (230–400 mesh). The column was eluted with hexane/ethyl acetate (90:10), (80:20) and (70:30). The fractions from gradient (70:30) were dried to afford a yellowish-brown-colored compound which was found to give single spot in TLC and was named as BV4 fraction (40 mg) (Figure 3).

### 2.3. Recording of Spectral Data

The chemical characterization and structure interpretation of the pure isolated compounds was carried out with nuclear magnetic resonance (NMR), Fourier Transform Infra-red (FTIR) and mass spectroscopy.

### 2.4. Nuclear Magnetic Resonance Spectroscopy 

NMR spectroscopy helps determining the unique structure of a compound by identifying the carbon-hydrogen framework. Samples for NMR were prepared in deuterated methanol (CD_3_OD), in standard (15 cm × 5 mm) NMR quartz tubes. ^1^H-NMR and ^13^C-NMR techniques were used. The NMR spectra were recorded on Brucker Avance-300.

### 2.5. Fourier Transform Infra-Red Spectroscopy

FTIR spectroscopy identifies the functional groups as these generate characteristic bands indicating intensity and position (frequency). The units of frequency used are reciprocal centimeters abbreviated as cm^−1^.

### 2.6. Mass Spectroscopy

Mass spectroscopy determines molecular weight of a compound. The compound (2 mg) was sonicated with HPLC grade acetonitrile and water (1:1; 15 min at room temperature) and the resulting solution was injected (20 µL) into the mass spectrophotometer (Bruker) to record the mass spectrum. 

### 2.7. Antioxidant Capacity

#### 2.7.1. ABTS Radical Scavenging Assay 

Method given by Re et al. [28] was used for determination of the electron and hydrogen donating capacity of isolated compound fractions of *B. variegata* bark using ABTS^+^ radical as a substrate. 2,2-azinobis-3-ethyl-benzothiazoline-6-sulfonic acid (ABTS) radical cation, which has blue-green chromophore absorption, is produced by oxidation of ABTS with potassium persulfate prior to the addition of extract. The antioxidant activity of the fractions was determined by the decolorization of the ABTS, by measuring the reduction of the radical cation as the percentage inhibition of absorbance at 734 nm. BHT (butylated hydroxytoluene) was used as the positive control. All determinations were carried out in triplicate.

Percentage inhibition of ABTS radical cations was calculated using the following equation:

(1) $$\%\;\mathrm{Inhibition}=\left[\frac{\left(\mathrm{Absorbance}\;\mathrm{of}\;\mathrm{control}-\mathrm{Absorbance}\;\mathrm{of}\;\mathrm{Sample}\right)}{\mathrm{Absorbance}\;\mathrm{of}\;\mathrm{control}}\right]\times 100$$

#### 2.7.2. DPPH Free Radical Scavenging Assay

Method given by Blois [29] was utilized for determination of hydrogen donating capacity of isolated compounds of *B. variegata* bark using 2,2’-diphenyl-1-picrylhydrazyl radical as a substrate. The methanolic solution of DPPH is a stable radical with peak absorbance at 517 nm. The absorbance disappears due to reduction of 2,2’-diphenyl-1-picrylhydrazyl radical (purple-colored solution) to 2,2’-diphenyl-1-picryl hydrazine (yellow-colored solution), a diamagnetic stable molecule due to the presence of an antioxidant or reaction with free radical species. BHT was used as positive control. All tests were performed in triplicate. Percentage inhibition of DPPH radicals was calculated using the following equation:

(2) $$\%\;\mathrm{Inhibition}=\left[\frac{\left(\mathrm{Absorbance}\;\mathrm{of}\;\mathrm{control}-\mathrm{Absorbance}\;\mathrm{of}\;\mathrm{Sample}\right)}{\mathrm{Absorbance}\;\mathrm{of}\;\mathrm{control}}\right]\times 100$$

### 2.8. Cytotoxic Activity Assay

The extent of cytotoxicity of the isolated compounds in cancerous cells was obtained using MTT dye (3-(4,5-dimethylthiazol-2-yl)-2,5-diphenyl tetrazolium bromide) reduction assay, as proposed by Igarashi and Miyazawa [30]. The cell lines C6 and MCF-7 were maintained on DMEM (Dulbecco’s modified Eagle’s minimal essential medium) and HCT-15 cell line was maintained on RPMI Medium (Rosewell Park Memorial Institute Medium) at 37 °C and humid environment containing 5% CO_2_. The cells (2000 cells/well in 96 well plate), treated with different concentrations (31.2, 62.5,125, 250,500 and 1000 µg/mL) for 24 h, were incubated with 10μL of MTT (37 °C, 2 h). After the incubation, media in the cells was aspirated, followed by addition of DMSO (100 μL) in each well. The absorbance was recorded in a micro titer plate reader at 570 nm. The control cells optical density (OD) was fixed 100% viability and thus the per cent cytotoxicity of test compounds in the other treatment groups was calculated as follows:

(3) $$\%\mathrm{Cytotoxicity}=\left[1-\frac{\mathrm{Sample}\;\mathrm{OD}}{\mathrm{Control}\;\mathrm{OD}}\right]\times 100\%$$

### 2.9. Statistical Analysis

All data were subjected to one-way analysis of variance (ANOVA) expressed as the mean ± standard deviation of three replicates. The difference among the average values was compared using Tukey’s HSD test (honestly significant difference; level of significance *p* ≤ 0.5). Best fit method and regression equation was used for calculating IC_50_ values (concentration of the fraction at which they were able to inhibit 50% of the radicals) and GI_50_ values (the concentration of the extract able to hinder the cell growth to 50%). 

## 3. Results

### 3.1. Structural Elucidation of BV1

BV1 was a yellow amorphous powder. The ^1^H NMR spectrum provided a set of broad singlets at δ 6.40 and δ 6.19 assigning to H-8 and H-6 protons respectively. The set of doublets at *δ* 8.10 and *δ* 6.91 corresponded to protons at H-2’, H-6’ and H-3’, H-5’ respectively. The ^13^C NMR spectrum revealed six oxygenated carbons at *δ* 166.0, 163.0, 161.0, 158.7, 131.1, and 124.2 assignable to C-7, C-5 C-4’, C-8a, C-2, C-3, respectively. The signal at *δ* 177.8 exposed the presence of carbonyl group at C-4, thus confirming the flavonol type of skeleton (Table 1). The IR absorption spectrum displayed the absorption peaks at 3421.48 cm^−1^ (OH stretching, hydrogen bonded) and 2916.17 cm^−1^ (C-H sp^2^ carbon). The peak at 1174.57 cm^−1^ belongs to the C-O group and similarly, 1612.38 cm^−1^ belongs to the C=C (aromatic). The positive ESI-QTOF-MS revealed a molecular peak at *m*/*z* 287.044 [M+H]^+^ which indicated the molecular formula C_15_H_10_O_6_. Thus on the basis of above spectral data and comparison with previously known spectral values [31], the structure of fraction BV1 was assigned as 3,5,7-trihydroxy-2-(4-hydroxyphenyl)-4*H*-1-benzopyran-4-one (Kaempferol).

### 3.2. Structural Elucidation of BV2

The compound BV2 was a white powder. The ^1^H NMR spectrum of BV2 revealed two methyl singlets at δ 0.71, and δ 1.02; three methyl doublets at δ 0.80, δ 0.82, and δ 0.93; and a methyl triplet at δ 0.82. BV2 also had protons at δ 5.14 and δ 5.36, thus suggesting the protons which correspond to trisubstituted and a disubstituted olefinic bond. The proton appeared as a triplet of doublet of doublets at δ 3.52; correspond to the H-3 of a sterol moiety. The spectral data supports a sterol skeleton with a hydroxyl group at C-3 position and two double bonds at C-5/C-6 and C-20/C-21 along with six methyl groups (Table 2).

The IR absorption spectrum presented absorption peaks at 2960.53 cm^−1^ (C-H sp2 carbon) and 2854.45 cm^−1^ (C-H stretching sp3 carbon); 1458.08 cm^−1^ (CH2 bending); 1261.36 cm1 (C-O group). The positive ESI-QTOF-MS indicated molecular peak at m/z 413.294 [M+H]^+^ (calculated 412.690), indicating the molecular formula C_29_H_48_O. The above spectral data is in comparison with previously known spectral values of stigmaserol [32,33,34], thus, BV2 was assigned the structure of the known compound, stigmasterol ((3S,8S,9S,10R,13R,14S,17R)-17-[(E,2R,5S)-5-ethyl-6-methylhept-3-en-2-yl]-10,13-dimethyl-2,3,4,7,8,9,11,12,14,15,16,17-dodecahydro-1H-cyclopenta[a]phenanthren-3-ol).

### 3.3. Structural Elucidation of BV3 

The fraction BV3 was obtained as a brown solid mass. The ^1^H NMR spectrum revealed signals at *δ* 7.42 for C-5 and C-6 protons each and at *δ* 6.80, suggesting a 1,3,4-trisubstituted benzene ring. The slightly down fielded signal at *δ* 3.84 indicated methoxy group in the structure. The ^13^C NMR spectrum revealed seven carbons with a signal at *δ* 167.8 assignable to carboxyl carbon (-COO). The signals at *δ* 122.5, 121.5, 116.3, and 114.8 corresponded to C-6, C-1, C-2, and C-5 carbons respectively. Similarly, signals at *δ* 145.1 and 150.6 represented C-3 and C-4 aromatic carbons bearing hydroxyl groups. The methoxy group at C-1’ yielded a signal at *δ* 51.2 (Table 3). 

The IR absorption spectrum showed peaks mainly in the region of 3600–3200 cm^−1^ (O-H group), 1300–1000 cm^−1^ (C-O group). A C-H stretching of sp^3^ carbon was corresponded to the bands at 2954.74 cm^−1^ and 2925.81 cm^−1^. The peak at 1697.24 cm^−1^ corresponded to C=O and the peaks at 1604.66 cm^−1^ and 1456.16 cm^−1^ suggested C=C of aromatic ring. Thus, fraction BV3 was recognized as 3,4-dihydroxybenzoic acid methylester (protocatechuic acid methyl ester) with molecular formula C_8_H_8_O_4_, based on the spectral data.

### 3.4. Structural Elucidation of BV4

The fraction BV4 was a yellowish-brown powder. The ^1^H NMR spectrum showed signals at *δ* 7.43, 7.43 and 6.81 indicating a 1,3,4-trisubstituted benzene ring. ^13^C NMR spectrum revealed seven carbons with a signal at *δ* 169.2 assignable to carboxyl carbon (COOH). Similarly, four signals at *δ* 122.9, 122.0, 116.7, and 114.7 corresponded to C-6, C-1, C-2, and C-5 carbons respectively. C-3 and C-4 aromatic carbons bearing hydroxyl groups corresponded to the signals at *δ* 145.0 and 150.5 (Table 4).

The IR spectrum showed peaks in 3600–3200 cm^−1^ region (O-H group of the phenyl ring as well as of the carboxylic acid) and in the 1300-1000 cm^−1^ region (C-O group). There was C-H stretching of sp^3^ carbon revealed by the bands at 2954.74 cm^−1^, 2929.67 cm^−1^. A peak at 1701.10 cm^−1^ suggested C=O and a peak at 1608.52 cm^−1^ correspond to C=C of aromatic ring. The C-O stretching vibration for acid appeared in the region of 1292–1230 cm^−1^. The peak at 943.13 cm^−1^ suggested hydrogen bonded OH out of plane bending vibration.

Thus, fraction BV4 was recognized as 3,4-dihydroxybenzoic acid (protocatechuic acid) with molecular formula C_7_H_6_O_4_, based on data that matched with the reported spectral data [35].

### 3.5. Antioxidant Activity

**Figure 4:**
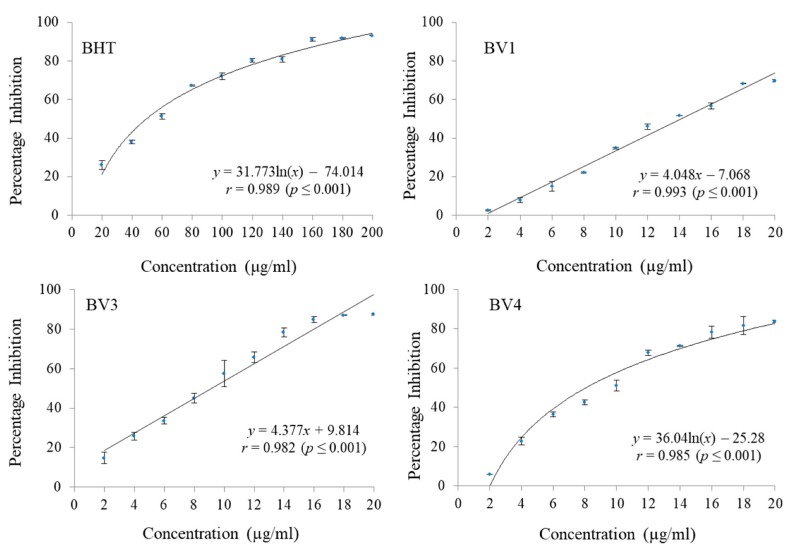
The ABTS radical scavenging activities of standard reference compound BHT and compounds isolated from *Bauhinia variegata* L. stem bark. BHT = butylated hydroxytoluene (standard reference compound); BV1 = kaempferol; BV3 = protocatechuic acid methyl ester; BV4 = protocatechuic acid.

**Figure 5:**
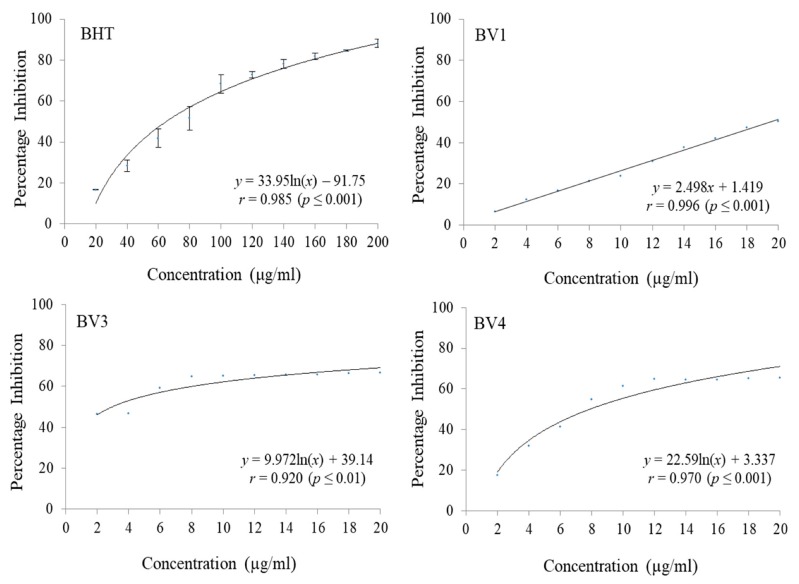
The DPPH radical scavenging activities of standard reference compound BHT and compounds isolated from *Bauhinia variegata* L. stem bark. BHT = butylated hydroxytoluene (standard reference compound); BV1 = kaempferol; BV3 = protocatechuic acid methyl ester; BV4 = protocatechuic acid.

It was found that BV3 was able to scavenge 87.38% ABTS radicals followed by BV4 (83.82% scavenging) and BV1 (69.73% scavenging) at the maximum tested concentration (20 µg/mL) respectively. At the concentrations ranging from 2 µg/mL to 20 µg/mL, BV2 did not show any notable ABTS radical scavenging activity (Figure 4 and Table 5). The fractions were also found to be potent inhibitors of DPPH free radicals with BV1 having 50.51% DPPH radical inhibition at a concentration of 20 µg/mL. BV3 scavenged 66.45% DPPH radicals and BV4 was able to scavenge 65.33% DPPH radicals at the same concentration (Figure 5 and Table 6). BV2 was not found to possess any considerable DPPH radial scavenging activity at the highest tested concentration. BHT was used a standard reference antioxidant compound which did not show considerable activity at the concentration range 2 µg/mL to 20 µg/mL and to calculate its IC_50_, it was tested at a concentration range of 20 µg/mL to 200 µg/mL. In both ABTS and DPPH radical scavenging assays, the compounds isolated from *B. variegata* exhibited a lower IC_50_ than BHT.

### 3.6. Cytotoxic Activity

**Figure 6:**
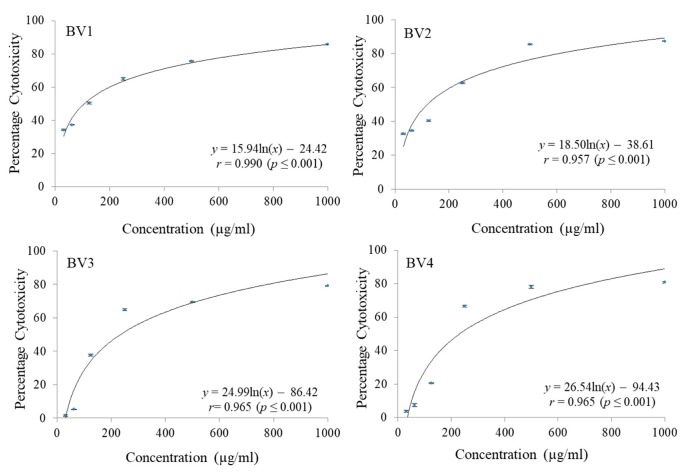
The percentage cytotoxic activity of compounds isolated from *Bauhinia variegata* L. bark against C6 cell line. BV1 = kaempferol; BV2 = stigmasterol; BV3 = protocatechuic acid methyl ester; BV4 = protocatechuic acid.

**Figure 7:**
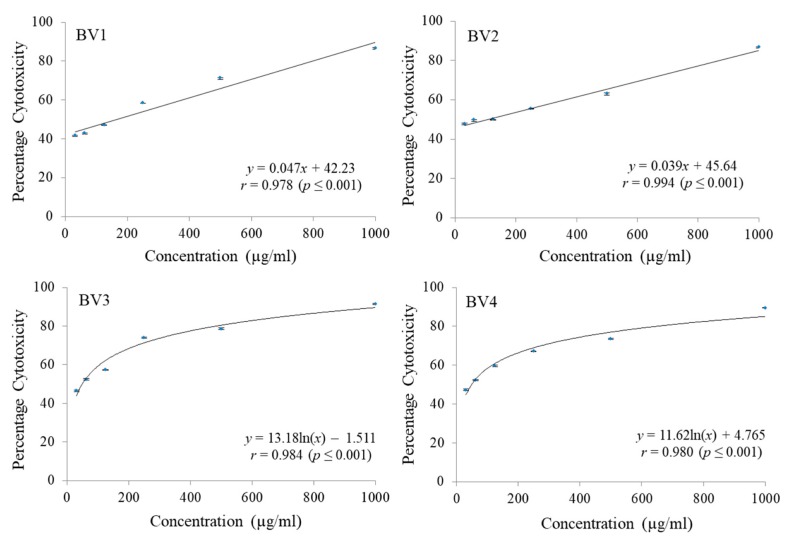
The percentage cytotoxic activity of compounds isolated from *Bauhinia variegata* L. bark against MCF-7 cell line. BV1 = kaempferol; BV2 = stigmasterol; BV3 = protocatechuic acid methyl ester; BV4 = protocatechuic acid.

**Figure 8:**
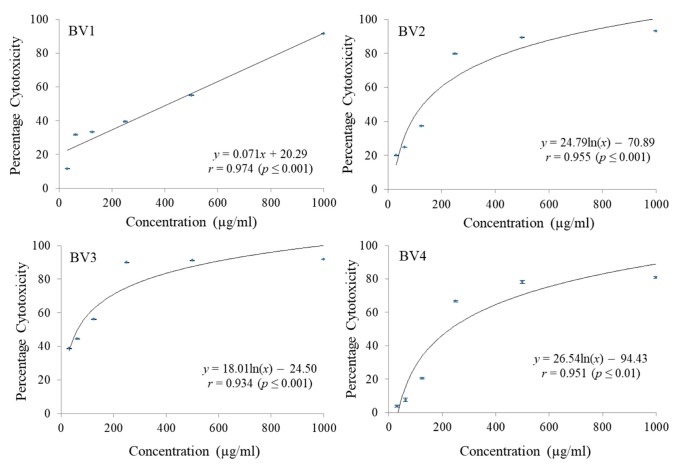
The percentage cytotoxic activity of compounds isolated from *Bauhinia variegata* L. bark against HCT-15 cell line. BV1 = kaempferol; BV2 = stigmasterol; BV3 = protocatechuic acid methyl ester; BV4 = protocatechuic acid.

The cytotoxicity of pure fractions obtained from column chromatography of MEB extract were checked against C-6 glioma rat brain, MCF-7 breast cancer and HCT-15 colon cancer cell lines employing MTT cell viability assay. A significant reduction in dose dependent manner was noticed for different fractions. Different concentrations ranging from 31.25 µg/mL to 1000 µg/mL were checked. Maximum reduction in C-6 cell proliferation was shown by BV1 followed by BV2 and BV3 both having almost the same GI_50_ values. The fraction BV4 was found to have the maximum GI_50_ in case of C6 cell line (Figure 6 and Table 7). The percentage cytotoxicity against MCF-7 cell line was also observed to increase with increase in concentration of fractions being tested and in terms of GI_50_ values, maximum cytotoxiciy was shown by BV4 followed by BV3, BV2, and BV1 (Figure 7 and Table 7). Similarly, in case of HCT-15 cell line, maximum cytotoxicity against the cancerous cells was observed in case of BV3 followed by BV2, BV4 and BV1 having the maximum GI_50_ value (Figure 8 and Table 7).

## 4. Discussion

Natural plant products have traditionally been an imperative source of lead molecules in drug discovery. Despite this fact, the interest of the pharmaceutical industries in natural products has declined in the past. There has been observed a switching away from natural products to combinatorial chemistry for drug development [36]. The reason behind this change seems to be the fact that chemical agents were far easier to synthesize, to get patented and furthermore these ensured greater profitability for industries. However, synthetic drug side effects became a major health concern leading to withdrawal of certain drugs from the market.

Earlier, the isolation and characterization of bioactive compounds from different plant parts was a long, gradual process, contingent upon the convolution of the study. Nowadays, however, pace of bioassay-guided fractionation has escalated by use of instrumentation like higher magnetic field-strength NMR, high-performance liquid chromatography (HPLC), liquid chromatography mass spectroscopy (LC-MS), etc. [37]. Drug discovery from natural sources is gaining pace in correspondence with rise in identification of new lead compounds.

The chemical composition of the plants belonging to genus *Bauhinia* shows a high proportion of flavonoids, terpenes, steroids and alkaloids. Isolation of Lupeol, 5, 7-dimethoxyflavonone-4–O–α– L–rhamnopyranosyl-β-D-glucopyranoside, β-sitosterol, kaempferol-3- glucoside from the stem of the have been reported [38]. In the present study, pure fractions isolated from MEB through silica gel column chromatography, i.e., BV1, BV2, BV3 and BV4, were characterized as kaempferol, stigmasterol, protocatechuic acid-methyl ester (PCA-ME) and protocatechuic acid (PCA) respectively, and were subjected to analysis of their antioxidant potential and cytotoxic activity against different cell lines.

Kaempferol has been reported to have very significant antioxidant activity [39]. It is a flavonoid frequently found in plant-based foods as well as in plant parts utilized in folk medicine. It has a double bond at C2-C3 and an oxo group at C4, and hydroxyl groups at C3, C5 and C4’, which provide the structural bases for antioxidant activity [40,41,42]. In the present study, it significantly inhibited ABTS and DPPH free radicals, which is in conjugation with a number of studies reporting that kaempferol, and its glycosides, and various plants parts having kaempferol possess antioxidative potential [43]. Stigmasterol, known as the Wulzen anti-stiffness factor, belongs to the group of plant sterols having similarity to animal cholesterol. The manufacture of vitamin D3 and semi-synthetic progesterone, which is a valuable human hormone, involves stigmasterol as a precursor [44]. In the present investigation, when analyzed for its activity against ABTS and DPPH free radicals, it did not show any considerable antioxidative activity. Similar results were obtained by Shanthakumar et al. [45] where stigmasterol was found to possess a very high IC_50_ of 220µg/mL against DPPH radicals.

Protocatechuic acid has wide natural distribution and shares structural similarity with caffeic acid, vanillic acid, gallic acid, syringic acid which are well acknowledged antioxidants [46]. In the present investigation, both PCA and PCA-ME showed considerable antioxidant and cytotoxic activities. The PCA-ME had a higher potential to scavenge free radicals as compared to PCA. In case of ABTS assay, PCA-ME possessed an IC_50_ of 8.76 ± 1.23 µg/mL as compared to PCA having an IC_50_ of 9.39 ± 0.45 µg/mL. Similar was the case with DPPH assay where the IC_50_ values were 4.46 ± 0.21 µg/mL and 7.28 ± 1.64 µg/mL for PCA-ME and PCA respectively.

Literature reports also suggest lower radical scavenging activity of PCA compared to PCA-ME in alcoholic solvents. In an experiment carried out by Saito and Kwabata [47], it was found that PCAME scavenged 5 equivalents of DPPH radical in contrast to 2 equivalents of radical scavenging by PCA in methanol in 30 min. Further, kaempferol, stigmasterol, PCA-ME and PCA were subjected to analysis of their cytotoxic prospective against C6, MCF-7 and HCT-15 cell lines. A chemically induced rat brain tumor was used to clone C6 rat glioma cell line, and is categorized as an undifferentiated astrocytic cell type [48]. It is extensively used in in vitro models to study glial cell properties. The MCF-7 has been taken from a patient’s pleural effusion suffering with metastatic breast cancer [49]. In the present investigation, kaempferol exhibited dose dependent increase in the cytotoxic capacity against different cell lines under study. Kaempferol showed maximum cytotoxicity against C-6 cell line, followed by MCF-7 and HCT-15 cell line. Earlier reports by Diantini et al. [50] stated that kaempferol-3*-O*-rhamnoside hinders the proliferation of MCF-7 cells dose-dependent mode and also promotes apoptosis. Among A549 and MCF-7 cells, induction of MAPK (mitogen-activated protein kinase) has been considered to be an essential factor in kaempferol initiated apoptosis. Also, the MAPK activation mediated by kaempferol may avoid impairment to DNA leading to cell transformation. Kaempferol was further found to increase expression of haeme oxygenase (HO)-1 gene, resulting in a surge in the antioxidant capacity of cells [51]. In HeLa cells, kaempferol blocked the glucose cellular intake [52]. A cytostatic activity of 24.8–64.7 μM by kaempferol was observed in PC3, HeLa and K562 human cancer cells [53]. Luo et al. [54] reported kaempferol to initiate activation of p53 thus inducing apoptosis in ovarian cancer cells. It has also inhibited quinine reductase 2 (IC_50_ 33.6 μM) for NF-κB activity [55]. Similarly, Kim and Choi [56] reported kaempferol to inhibit growth of MCF-7 cells via regulating cell cycle and apoptosis-related genes. Combining kaempferol with chemotherapeutic drugs has produced better therapeutic effects than the drugs alone, and this combination also reduces the toxicity of chemotherapeutic drugs [57]. Imran et al. [58] reported that kaempferol-rich food decreases the risk of development of skin, liver, and colon cancers via apoptosis, cell cycle arrest, downregulating epithelial-mesenchymal transition (EMT) related markers as well as the phosphoinositide 3-kinase/protein kinase B signaling pathways. They suggested that cancer fighting properties of kaempferol are extraordinary as it specifically targets cancerous cells without damaging healthy cells.

In case of stigmasterol, when evaluated for cytotoxic potential, it was observed that it showed moderate activity against the cell lines under investigation showing GI_50_ values of 173.97 ± 0.81 µg/mL, 132.01 ± 0.55 µg/mL, 151.20 ± 0.74 µg/mL for C-6, MCF-7 and HCT-15 cell lines respectively. Malikova et al. [59] established that stigmasterol showed very weak to undetectable activity against the T-lymphoblastic leukaemia CEM, MCF-7 (breast carcinoma), A-549 (lung carcinoma), K562 (chronic myeloid leukaemia), HeLa (cervical carcinoma), G361 (malignant melanoma) and HOS (osteosarcoma) cell lines and had a moderate activity against multiple myeloma RPMI 8226 cell line. However, in a study conducted by Ali et al. [60], a reduction in size of tumor and number of papillomas was observed with an oral administration of stigmasterol to Swiss albino mice in which had skin tumors induced through topical application of 7,12-dimethylbenz[a]anthracene (DMBA).

PCA have shown considerable cytotoxic potential against MCF-7 cell line (GI_50_ 49.14 ± 0.23 µg/mL), while PCA-ME have shown significant cytotoxic activity against MCF-7 (GI_50_ 54.18 ± 0.16 µg/mL) as well as against HCT-15 cell line (GI_50_ 92.17 ± 1.17 µg/mL). The mechanism of cytotoxic action of PCA has been proposed to be associated with antioxidant activity. It can influence metabolism phases 1 and 2 of various carcinogens thus blocking specific binding sites of the carcinogens with the DNA. This leads to the prevention of adduct formation that could have resulted into mutations and neoplastic transformations [61]. Various researchers have reported PCA to have significant antioxidant, antibacterial, anti-inflammatory, analgesic and antiseptic properties [62,63]. In a study conducted by Bullo et al. [64], PCA and chlorogenic acid (CA) were loaded into an anticancer nanocarrier graphene oxide-polyethylene glycol (GOPEG) and tested in phosphate buffer saline to assess the release properties and also tested against HEPG2 (liver cancer), HT-29 cells (human colon cancer) and normal 3T3 cells (fibroblast cells). It was found that PCA and CA were released in a sustained manner, having strong activity against cancerous cells and biocompatibility with normal cells.

The phytochemical screening of *B. variegata* has shown the occurrence of flavonoids, terpenoids, reducing sugars, steroids, saponins, tannins, and cardiac glycosides [65]. In earlier studies, *B. variegata* has depicted chemopreventive activity against liver tumors induced by *N*-nitrosodiethylamine and human cancer cell lines. It was found that orally administrating ethanol extract of *B. variegata* suppressed liver tumor. A decrease in serum glutamate pyruvate transaminase, lipid peroxidase, total bilirubin, glutathione peroxidase and glutathione *S*-transferase levels was observed. Further, an increase in superoxide dismutase, catalase and total proteins was also observed [66]. A phytochemical investigation of the polar fractions of methanol extract of *B. variegata* bark revealed that the fractions were rich in phenolic and flavonoid content and a statistically significant correlation was observed between phenolic/flavonoid content and the antioxidant activity of the fractions [67]. The essential oil of *B. variegata* flowers has been reported to contain a substantial amount of nerolidol, α-bisabolol and β-bisabolene which have significant medicinal properties [68].

## 5. Conclusions

A number of higher plant species are used in traditional medicine have not yet screened for the presence of powerful bioactive compounds. Different plant parts of *B. variegata* L. being used in traditional medicine calls for the chemotaxonomic identification. The current study revealed presence of strong antioxidant and cytotoxic phytochemicals (kaempferol, stigmasterol, protocatechuic acid methyl ester and protocatechuic acid) in the bark. It is concluded that *B. variegata* L. can be a potential addition in pharmaceutical products for the improvement of human health by contributing in the antioxidant defense system fighting against the production of free radicals.

## Figures and Tables

**Table 1:** The ^1^H and ^13^C NMR spectral data (recorded in CD_3_OD) of BV1 fraction isolated from *Bauhinia variegata* L. bark.

Carbon No.	^1^H NMR (δppm)	^13^C NMR (δppm)
2	-	131.1
3	-	124.2
4	-	177.8 (C=O)
5	-	163.0
6	6.19 (1H, br s)	99.7 (CH)
7	-	166.0
8	6.40 (1H, br s)	94.9 (CH)
9	-	158.7
10	-	105.0
1’	-	124.2
2’	8.10 (2H, d, *J* = 8.8 Hz)	131.1 (CH)
3’	6.91 (2H, dd, *J* = 8.8 Hz)	116.7 (CH)
4’	-	161.0
5’	6.91 (2H, dd, *J* = 8.8 Hz)	116.7 (CH)
6’	8.10 (2H, d, *J* = 8.8 Hz)	131.1 (CH)

H = hydrogen; C = C arbon; δppm = chemical shift; br = broad signal; s = singlet; d = doublet; dd = doublet of doublets; *J* = coupling constant; Hz = hertz; NMR = nuclear magnetic rasonance; CD_3_OD = Deuterated methanol.

**Table 2:** The ^1^H and ^13^C NMR spectral data (recorded in CD_3_OD) of BV2 fraction isolated from *Bauhinia variegata* L. bark.

Carbon No.	^1^H NMR (δppm)	^13^C NMR (δppm)
1	-	37.6
2	-	32.0
3	3.52	72.2 (CH)
4	-	42.7
5	-	141.1
6	5.36 (H, dd)	122.1 (CH)
7	-	32.0
8	-	32.3
9	-	50.5
10	-	36.5
11	-	21.4
12	-	37.6
13	-	42.7
14	-	57.1
15	-	24.7
16	-	29.5
17	-	56.4
18	0.71	12.3 (CH)
19	0.93	19.4
20	-	40.1
21	1.02 (H, d)	21.4 (CH)
22	5.36	138.7 (CH)
23	-	129.6
24	-	50.5
25	-	32.0
26	0.82 (2H, d)	19.1 (CH_3_)
27	0.80 (2H, d)	21.4 (CH_3_)
28	-	24.7 (CH_3_)
29	0.82 (2H, d)	12.2 (CH_3_)

H = hydrogen; C = Carbon; δppm = chemical shift; br = broad signal; s = singlet; d = doublet; dd=doublet of doublets; *J* = coupling constant; Hz = hertz; NMR = nuclear magnetic rasonance; CD_3_OD = Deuterated methanol.

**Table 3:** The ^1^H and ^13^C NMR spectral data (recorded in CD_3_OD) of BV3 fraction isolated from *Bauhinia variegata* L. bark.

Carbon No.	^1^H NMR (δppm)	^13^C NMR (δppm)
1	-	121.5
2	6.80 (H, d, *J* = 8.82Hz)	114.8 (CH)
3	-	145.1
4	-	150.6
5	7.42 (H, d, *J* = 10.8 Hz)	116.3 (CH)
6	7.42 (H, d, *J* = 10.8 Hz)	122.5 (CH)
COO	-	167.8
OCH_3_	3.84	51.2

H = hydrogen; C = Carbon; δppm = chemical shift; br = broad signal; s = singlet; d = doublet; dd = doublet of doublets; *J* = coupling constant; Hz = hertz; NMR = nuclear magnetic rasonance; CD_3_OD = Deuterated methanol; COO = carboxyl carbon group; OCH_3_ = methoxy group.

**Table 4:** The ^1^H and ^13^C NMR spectral data (recorded in CD_3_OD) of BV4 fraction isolated from *Bauhinia variegata* L. bark.

Carbon No.	^1^H NMR (δppm)	^13^C NMR (δppm)
1	-	122.9
2	7.43 (H, s)	116.7 (CH)
3	-	145.0
4	-	150.5
5	6.81 (H, d, *J* = 8.6Hz)	114.7 (CH)
6	7.43 (H, d, *J* = 8.1Hz)	122.9 (CH)
COOH	-	169.2

H = hydrogen; C = Carbon; δppm = chemical shift; br = broad signal; s = singlet; d = doublet; dd = doublet of doublets; *J* = coupling constant; Hz = hertz; NMR = nuclear magnetic rasonance; CD_3_OD = Deuterated methanol; COOH = carboxyl group.

**Table 5:** IC_50_, one-way ANOVA F-ratio and HSD values of BHT and compounds isolated from *Bauhinia variegata* L. stem bark in ABTS radical scavenging assay.

Parameter	BHT	BV1	BV3	BV4
**IC_50_ (µg/mL)**	58.48 ± 0.64	13.45 ± 0.19	8.77 ± 1.23	9.39 ± 0.45
**F ratio (df = 9,20)**	363.11 *	481.19 *	91.77 *	157.07 *
**HSD**	6.19	5.63	14.11	10.71

df = degree of freedom; * Significant at *p* ≤ 0.05.; IC_50_ = 50% inhibition concentration; ANOVA = Analysis of variance; HSD = Honestly significant difference; BHT = butylated hydroxytoluene (standard reference compound); BV1 = kaempferol; BV3 = protocatechuic acid methyl ester; BV4 = protocatechuic acid. Values are mean ± standard deviation, *n* = 3.

**Table 6:** IC_50_, one-way ANOVA F-ratio and HSD values of BHT and compounds isolated from *Bauhinia variegata* L. stem bark in DPPH radical scavenging assay.

Parameter	BHT	BV1	BV3	BV4
**IC_50_ (µg/mL)**	76.16 ± 2.63	19.44 ± 0.58	4.46 ± 0.21	7.28 ± 1.64
**F ratio (df = 9,20)**	69.73 *	185.39 *	75.28 *	216.97 *
**HSD**	1.14	5.58	4.58	5.80

df = degree of freedom; * Significant at *p* ≤ 0.05.; IC_50_ = 50% inhibition concentration, lower IC_50_ means higher antioxidant activity; ANOVA = Analysis of variance; HSD = Honestly significant difference; BHT = butylated hydroxytoluene (standard reference compound); BV1 = kaempferol; BV3 = protocatechuic acid methyl ester; BV4=protocatechuic acid. Values are mean ± standard deviation, *n* = 3.

**Table 7:** GI_50_, one-way ANOVA F-ratio and HSD values of cytotoxic activity of compounds isolated from *Bauhinia variegata* L. bark against C6, MCF-7 and HCT-15 cell lines.

Cell Line	Compound			
	BV1	BV2	BV3	BV4
**C6**				
GI_50_ (µg/mL)	119.38 ± 0.34	173.97 ± 0.81	173.67 ± 0.57	203.16 ± 0.27
F ratio (df = 9,20)	8310.21 *	15,198.30 *	21,654.78 *	8804.11 *
HSD	1.09	0.99	1.08	1.83
**MCF-7**				
GI_50_ (µg/mL)	151.51 ± 0.22	132.02 ± 0.55	54.18 ± 0.16	49.15 ± 0.23
F ratio (df = 9,20)	9249.42 *	3776.94 *	7560.71 *	7280.99 *
HSD	0.89	1.14	0.95	0.86
**HCT-15**				
GI_50_ (µg/mL)	419.83 ± 0.41	151.20 ± 0.74	92.17 ± 1.17	203.15 ± 0.45
F ratio (df = 9,20)	44,831.82 *	130,522.70 *	64,468.07 *	64,642.00 *
HSD	0.97	0.44	0.48	0.76

df = degree of freedom; * significant at *p* ≤ 0.05.; GI_50_ = 50% growth inhibition, lower GI_50_ means higher cytotoxic activity; ANOVA = Analysis of variance; HSD = Honestly significant difference; BV1 = kaempferol; BV2 = stigmasterol; BV3 = protocatechuic acid methyl ester; BV4 = protocatechuic acid. Values are mean ± standard deviation, *n* = 3.
